# One Hundred and Sixty-One Days in the Life of the Homopandemicus in Serbia: The Contribution of Information Credibility and Alertness in Predicting Engagement in Protective Behaviors

**DOI:** 10.3389/fpsyg.2021.631791

**Published:** 2021-07-05

**Authors:** Žan Lep, Sandra Ilić, Predrag Teovanović, Kaja Hacin Beyazoglu, Kaja Damnjanović

**Affiliations:** ^1^Faculty of Arts, University of Ljubljana, Ljubljana, Slovenia; ^2^Laboratory for Experimental Psychology, Department of Psychology, Faculty of Philosophy, University of Belgrade, Belgrade, Serbia; ^3^Faculty for Special Education and Rehabilitation, University of Belgrade, Belgrade, Serbia

**Keywords:** COVID-19, coronavirus, credibility of information, alertness, self-protective behavior, protective behavior, pandemic stages, cross-sectional

## Abstract

COVID-19 pandemic is a long-lasting process associated with dynamic changes within society and in individual psychological responses. Effective communication of measures by credible sources throughout the epidemic is one of the crucial factors for the containment of the disease, and the official communication about pandemics is straightforwardly directed toward changes in behavior via engagement in (self-)protective measures. Calls for the adherence to these measures are aimed at the general population, but people's reactions to these calls vary depending on, for example, their individual differences in cognitive and emotional responses to the situation. The focus of our study was the general narrative about the epidemic as conveyed by both state officials and media outlets in times of decreased social contacts due to the quarantine, in which relying on these sources of information is even more pivotal. Our aim was to explore the stability of the proposed mediational model during the course of the epidemic in Serbia. In the model, we tested the relationship between perceived credibility of information (PCI) and two types of protective behavior—the actual self-protective behavior (ASPB) and the hypothetical protective behavior (HPB), as well as the potential mediating role of alertness in these relationships time-wise. A cross-sectional study (*N* = 10,782, female = 79.1%) was being administered daily during the first epidemic wave and in three more 2-week time frames during the second wave. Based on the variability of these measures during the first epidemic wave, three stages of psychological responses were mapped (acute, adaptation, and relaxation stage), which were observed, with some deviations, also in the second wave. The mediational model was relatively robust after the initial few weeks, but the strength of pairwise relationships was more changeable. With both types of protective behaviors, the predictive power of PCI was partially mediated through alertness. This suggests that, while individual differences in cognitive and affective responses are important, so is coherent, focused, and credible communication in all stages of the epidemic, which emphasizes the communality aspect of the social containment of the infection. Our findings can thus be valuable in informing the planning of effective future communication.

## Introduction

The epidemic of COVID-19 has been seriously affecting people's daily lives (Wang et al., 2020) and continues to do so. It has forced many countries around the world to adopt strict measures to contain the spread of the infection, including restricting social contacts, stopping public life, and keeping people under prolonged lock-downs. Studies conducted during previous epidemics, but also during the current COVID-19 pandemic, have shown that widespread occurrence of an infectious disease is indeed a source of stress (e.g., Cheng and Cheung, 2005; Casagrande et al., 2020; Kavčič et al., 2020; Petzold et al., 2020), not only because of growing concern and fear of the disease, but also because people have to adapt their lives to avoid becoming infected (e.g., Leung et al., 2005b). Moreover, global disease outbreaks are not one-time events, but longer-lasting processes associated with dynamic changes within society. Consequently, emotional and behavioral responses can change dramatically throughout the course of the outbreak, and especially after the occurrence of certain critical events or contextual changes (MHCC, 2012). At the same time, people respond differently to health threats and these individual differences may affect their health behavior (Brewer et al., 2007; Ferrer and Klein, 2015), which is crucial in curbing the spread of the disease.

While the effectiveness of different preventive measures and adherence to them on a population level has been quantitatively studied using mathematical models (e.g., Cacciapaglia et al., 2020; Cot et al., 2021), our focus was on the individual. We therefore focused on how perceptions related to COVID-19 and especially health-protective behavior differed from the confirmation of the first cases in Serbia in March through the official end of the COVID-19 associated state of emergency in May and beyond—until the end of the second epidemic wave in August. Furthermore, as epidemic outbreaks are related to significant and much needed behavioral changes that are only effective if their adoption is widespread (e.g., OECD, 2020), we were interested in whether the perception of the information received about COVID-19 is linked to protective behavior, and what is the role of individual differences in perceptions of the epidemic situation in this relationship.

### Credibility of Information

In order for people to behave appropriately and in accordance with the protective measures, each stage of the outbreak and the corresponding responses must be effectively communicated to the general public by both government and health officials, as well as the media (Reynolds and Seeger, 2014). In a situation of total lockdown, reduced social contact and increased risk of infection with the novel coronavirus, unknown to the general public, reliance on information from official sources is even more critical than in a non-crisis period (Austin et al., 2012; Chauhan and Hughes, 2017). From the beginning of the outbreak until its eventual control, but also during the follow-up period when the risk of a repeated outbreak is mitigated, public health authorities are expected to provide timely and accurate information and answers to the news media about the effects of the outbreak (Tumpey et al., 2018; WHO, 2018). These non-disputable facts provide the core of the information environment surrounding an epidemic. Indeed, numerous calls have been made about the importance of effective communication in fighting the infodemic (overflow of information with questionable validity; The Lancet, 2020).

One of the key features of this communication is the perceived credibility of the sources of information. Sources that are perceived as credible are more persuasive (Petty and Brinol, 2008; O'Keefe, 2016), and the credibility of information derives from the expertise and trustworthiness of the source (Van Bavel et al., 2020). Employing credible sources capable of sharing official public health facts has been shown to improve the effectiveness of public health messages in inducing behavioral change during epidemics (Lewandowsky et al., 2013; Greyling et al., 2016; Vijaykumar et al., 2018; Vinck et al., 2019; Van Bavel et al., 2020). In addition, credible information and public health messages from national leaders and health officials are required, and in line with the demands for effective communication, the media are a key factor in promoting healthy behavior (Sandman, 2009; Wakefield et al., 2010).

Due to the changing nature of the threatening event, people's trust in various institutions and information sources can change dynamically during the outbreak. Studies have shown that public support for the government during the H1N1 outbreak in Hong Kong in 2009 decreased over time (e.g., Yeung et al., 2017). Similarly, public trust in institutions in Switzerland decreased during the same outbreak (Bangerter et al., 2012). The trust in government and in medical institutions had a negative effect on anxiety, and at the same time it was found that the negative effect was stronger during the SARS outbreak than during the period thereafter (Cheung and Tse, 2008). A lack of trust in public health officials undermines the credibility of the information provided by officials, which may lead to lower levels of utilization of health services (Alsan and Wanamaker, 2018). In addition, alarming framing aimed at exaggerating the level of danger and intensive reporting in the mass media could trigger fear and hysteria (Van den Bulck and Custers, 2009). This, in turn, may limit the possibilities for mobilizing the public to adopt protective and health promoting behavior (Sherlaw and Raude, 2013). Additionally, negative emotions can also be amplified by prolonged exposure to negative reporting (Brug et al., 2004; Lau et al., 2010). Threatening and blaming discourse, negative allegations and the interference of personal emotions in the risk communication of pandemics undermine appropriate risk communication (Reynolds and Quinn Crouse, 2008). All this is contrary to the objectives of officials and health professionals and the general public interest.

As in many countries worldwide, the state reacted quickly in Serbia, and since mid-March, when the state of emergency was declared, numerous measures have been in force. Throughout this period, COVID-19 has been in the spotlight of coverage in various media. We focused on the official channels of pandemic-related communication, as the official media coverage was constant, relatively homogenized, and independent from the influences that less formal means of communication are subject to. Press conferences were held by appointed members of the crisis staff who informed public about the overall situation in the healthcare system (e.g., resources, designated COVID-19 hospitals, etc.), the official numbers of newly registered and total COVID-19 cases and deaths, both in Serbia and worldwide, as well as new and potential measures. They were broadcast daily during most of the first as well as the part of the second wave. During intermittent periods, press conferences were held a few days apart. Moreover, local and national television and radio stations, newspapers and news portals were, and still are, regularly reporting about coronavirus related numbers, stats, domestic, and international coronavirus-related news. In Serbia too, as is the case worldwide, the COVID-19 pandemic has established its association with every aspect of life, ranging across health, society, the economy, politics, the environment, sports, recitation, arts and culture, the media, innovation, and technology (Parvin et al., 2020). There was almost no news and stories unrelated to the epidemic covered by media outlets, even if dealing with some other, otherwise current topics. As different stages require different measures (WHO, 2018), we focused on the entire duration of the epidemic in Serbia, and examined how credible information sharing at different times could be efficient.

### Emotional Responses to Epidemic Situation and Alertness

As with perception of the information received, the extensive literature on past epidemics shows that emotional and behavioral responses change dramatically after the occurrence of certain critical events or after contextual changes, suggesting that these responses fluctuate across situations and over time (Theorell et al., 2005). The high increase in anxiety and similar emotional responses at the very beginning of the outbreak has been reported in studies conducted during the early stages of COVID-19 outbreak worldwide (Erceg et al., 2020; Garcia de Avila et al., 2020; Lep et al., 2020; Moghanibashi-Mansourieh, 2020; Özdin and Bayrak Özdin, 2020; Shevlin et al., 2020; Shiina et al., 2020; Wang et al., 2020). This abrupt increase in psychological distress was also observed during previous epidemics, followed by a decrease in intensity without any changes in the environment. For example, in a multiple-time-point study conducted during 4 weeks of the SARS outbreak in Hong Kong, the fluctuations in the state anxiety of the participants were measured over several points in time (Cheng and Cheung, 2005). The anxiety initially increased sharply and then gradually decreased at subsequent time points. In other words, although the outbreak continued to escalate and the number of deaths was still increasing, people's anxiety was lower at the last assessment than at the previous one. Apart from anxiety, various related perceptions may also be subject to the changes described. Yeung et al. (2017) conducted a longitudinal study during the H1N1 outbreak and found that the perceived severity of H1N1 virus infection and perceived susceptibility to infection decreased as the epidemic progressed, suggesting that the public gradually perceived a lower risk from the H1N1 virus. As the epidemic progresses, the psychological response adapts to the constantly changing circumstances, as with other universally stress-triggering events. When people endure psychological stress, they are usually first alarmed or struck by the occurrence of the dangerous event to which they react with highly intense emotional and behavioral reactions. When this initial impact is over, the next phase is less intense and involves resistance or possible recovery and adaptation in which the new daily routines take place, while the emotions relating to the epidemic are less sharp and dramatic. The last phase can be either exhaustion, burnout, or relaxation (Selye, 1946; Daly and Robinson, 2020).

However, Kiviniemi et al. (2018) emphasize the complex relationships between cognition and affect, especially in the field of health psychology. The concern or fear associated with health problems are often seen as emotional, unidimensional entities, although they can—to some extent—blend affect and cognition. In line with this, worry can be seen as an affect-laden cognitive process which, for example, involves affect occurring because of cognitions about a health problem or behavioral outcome. Therefore, in the present research we did not focus on traditional measures of anxiety, but rather observed the current alertness of the participants. The latter represents a more general pattern of people's cognitive propensity to observe and reflect on an epidemic and emotions that cause distress both in relation to the present moment, such as worry, and to future possibilities, such as fear of infection. Employing a serial cross-sectional design with daily measurements, our aim was to explore the differences in mean levels of perceived credibility of informational sources and alertness over the course of the pandemic, and how both could be utilized in predicting individual behavioral responses.

### Individual Responses and Protective Behavior

People's behavior and adherence to protective measures are fundamental factors in containing the disease, at least until the vaccine is available (Reynolds and Quinn Crouse, 2008; WHO, 2008; Tumpey et al., 2018; Van Bavel et al., 2020). Success in containing the spread of infection depends on people's compliance with the measures that may be under the influence of different individual characteristics and responses. Studies investigating factors positively influencing compliance with protective behavior showed an association between emotional and behavioral responses to the epidemics. A study conducted in 2009 during the H1N1 flu epidemic in the United States showed that affective variables, such as self-reported anxiety about the epidemic, mediate the likelihood that respondents will adopt protective behavior (Jones and Salathe, 2009). The results of the study, which included 10 cross-sectional surveys in Hong Kong, also showed a strong association between affective measures (i.e., affective measures of H1N1 risk perception) and adoption of protective behavior compared to cognitive measures of risk perception (Liao et al., 2014). Although this study showed that affective components consistently contribute to the adherence of protective behavior during an epidemic, other studies showed that this association remains positive in the early stages of the epidemic, but is usually not significant in the later stages (Leung et al., 2005a).

Moreover, the frequency of the use of different behavioral strategies during the epidemic changes over time, similar to the changes in emotional responses. During the H1N1 epidemic in Hong Kong various protective behaviors such as the use of facemasks or avoiding touching the face became increasingly less and less prevalent (Yeung et al., 2017). Similarly, during the SARS epidemic in Hong Kong, the practice of personal hygiene first increased and then slightly decreased, while avoidance strategies also increased strongly after the first assessment and then remained constant over time. At the same time, strategies related to the participants' search for information gradually decreased (Cheng and Cheung, 2005). Another study conducted during the initial phase of the SARS outbreak in Hong Kong showed that protective behaviors such as wearing a mask, washing hands, disinfecting at home, avoiding crowded places, and public transport increased significantly at the beginning, but only wearing a mask and washing hands remained at high levels, while a decrease was observed in all other protective behaviors (Lau et al., 2003). A study on public reactions during the early phase and peak of the H1N1 influenza in Greece also showed that during the peak of the pandemic compared to the early phase, participants reported adopting less protective behaviors (washing hands, avoiding crowds, asking a doctor for guidelines, etc.; Karademas et al., 2013). The authors concluded “such findings imply that perceptions, reactions and their relationships may change over the course of an epidemic influenza outbreak and may depend on several factors. Therefore, findings regarding public response at one epidemic phase may not apply to another.” (Karademas et al., 2013, p. 426). If affective responses and protective behavior change dynamically during the epidemic, the question remains whether the relationship between them is stable or does it also change during the epidemic.

### The Present Study

In sum, the aim of the present study was to examine the relationship between the perceived credibility of information (PCI), people's alertness, and their engagement in protective behavior over the course of an epidemic. Previous studies have shown that both alertness and perceived credibility are related to protective behavior (e.g., Cheung and Tse, 2008; Liao et al., 2010). However, the credibility of information might be directly related to engagement in protective behavior, or the credibility of information might spark or hinder alertness, which in turn would have an effect on engagement in protective behavior. Based on previous findings about the outbreak of COVID-19 in a culturally similar environment in Slovenia (Lep et al., 2020), where emotional responses to the epidemic were found to be related to the adoption of different protective behaviors, we tested the proposed mediational model and observed its stability over time as the pandemic progressed.

In addition, we also focused on changes in various psychological perceptions and reactions (e.g., participants' alertness, engagement in protective behaviors, and the perceived credibility of the information received) in view of the progression of the pandemic in Serbia, with the aim of gaining an insight into how these factors could be incorporated in policy-making to form interventions that encourage engagement in behaviors aimed at containing the spread of the disease while reducing negative emotional reactions, resulting in a shorter time frame of restrictive measures.

## Materials and Methods

### Sample

A cross-sectional study was conducted during the period of 24 weeks, from March 8th 2020 to August 15th 2020. All participants (*N* = 10,782, female = 79.1%) were legal adults −18 years old or older, native speakers of Serbo-Croatian language and Serbian residents, recruited via 1ka.si survey application using the Facebook advertising and snowball sampling method. They were between 18 and 92 years old (*M* = 39.98, *SD* = 13.31), and of adequate range in terms of educational level and the geographic distribution of population in the country. The sample size varied from day-to-day (between *n* = 13 and *n* = 396; 2 days with <10 participants were excluded from the analyses) and on weekly level (between *n* = 150 and *n* = 1,368; we excluded 2 weeks with <150 participants). Number of participants per day was larger initially, but it diminished over time when the epidemiological situation in Serbia improved (see Supplementary Table 2). Participants were not reimbursed for participation.

### Materials

The presented measures were part of a larger battery of tests used in the research on emotional and behavioral responses relative to trust in different sources of information during the first 48 h after the first confirmed case in Slovenia (Lep et al., 2020). For the present study we assessed alertness, actual self-protective behavior (ASPB) and hypothetical protective behavior (HPB), as well as the PCI about COVID-19 received by different sources. All measures were translated to Serbian by native speakers and, when needed, adapted to the Serbian context of the COVID-19 epidemic. The data on daily numbers of cases and deceased were obtained from the European Center for Disease Prevention and Control (ECDC, 2020).

#### Perceived Credibility of Information

Perceived credibility of information about the COVID-19 epidemic received from various information sources was measured using six items, rated on a five-point Likert-type scale (ranging from 1—not at all credible to 5—completely credible). The items referred to different available sources of COVID-19 information in the media. Participants were instructed to rate how credible they found the information they received about the coronavirus in the media from the following sources: the representatives of the Ministry of Health, Institute of Public Health of Serbia representatives, Medical chamber representatives, medical doctors, scientists, and journalists. Principal component analysis (PCA) revealed one principal component, which explained 70% of the variance in the dataset with an eigenvalue of 4.22. The reliability analysis indicated that the scale had very high internal consistency (α = 0.92). For complete scale see Supplementary Material.

#### Alertness

Five sets of two items, adapted from Li et al. (2020), were used to measure perceptions about and emotional responses to the epidemic, and subsequently aggregated into a cognitive-affective construct dubbed alertness. They referred to the degree of worry, fear of contracting the disease, possibility of limiting its spread, perceived severity, and the amount of thinking about the coronavirus both before and after the first confirmed case of the disease in Serbia. Participants rated each item on a six-point Likert-type scale. The scales were customized in accordance with the corresponding item content (e.g., 1—not at all worried, 6—very worried). A PCA run on all 10 items revealed three components explaining in total 74% of the variance in the data set. The first component pertained to the items measuring worry, fear of contracting the disease, perceived severity, and the amount of thinking about the coronavirus *after* the first confirmed case in Serbia. The third component referred to the same items rated in relation to the time *before* the first confirmed case in Serbia, while the second component pertained to the items measuring the possibility of containment of the disease, both before and after the first confirmed case. As the analysis showed two mirrored factors pertaining to both cognitive and emotional aspects of arousal, differing only in relation to the time the items referred to, we ran a second PCA on the items that loaded heavily on the first and third component. The analysis revealed two components differing by the time to which the items referred. The first component accounted for 57% of the variance with an eigenvalue of 4.59, and the second accounted for 17% of the variance with an eigenvalue of 1.34. For the purposes of this study, we used the four items comprising the first component, that is—alertness (after the first confirmed case). The scale exhibits very high internal consistency (α = 0.91), a single component explains 78% of the variance, and has an eigenvalue of 3.14. For complete scale see Supplementary Material.

#### Protective Behavior

To measure protective behavioral responses to the epidemic, we assessed engagement in ASPBs and HPBs. Actual self-protective behavior was measured using 10 items, rated on a three-point scales (with responses: does not apply, partly applies, and totally applies to me). Items were selected according to the guidelines regarding effective self-protective behaviors (e.g., washing hands thoroughly, not touching face, etc.) posted on websites of WHO and Institute of Public Health of Serbia. We have also added several behaviors, which were not labeled as recommended protective or preventive behaviors, but were registered as frequent in the first days of the epidemic (e.g., stockpiling food or medical supplies).

Hypothetical protective behavior was measured using six items, rated on a five-point Likert-type scale (1—I surely wouldn't, 5—I surely would). Items were selected based on recommendations given by the Ministry of Health and Institute of Public Health representatives regarding steps that should be taken if suspecting coronavirus infection (self-isolation, avoiding family members, skipping work, taking care of personal and the hygiene of home, calling and visiting community Health center).

Principal component analysis for the ASPB scale showed one principal component, which accounted for 46% of the variance, with an eigenvalue of 4.59. The scale had good internal consistency (α = 0.86). Principal component analysis of items comprising HPB showed two components: the first pertaining to protective behavior aimed at protecting others, explaining the 40% of the variance with an eigenvalue of 2.42, and the second pertaining to contacting a medical institution accounting for 18% of the variance. Further analysis showed that the answers to questions regarding contacting the medical institution varied based on the official guidelines on what one should do if one suspects they contracted the coronavirus. As the official instructions on whether one should *call* or *visit* the community Health center shifted at some point, so did the majority of respondent's answers to these two questions. Thus, these two items were omitted from further analyses. The HPB scale consisting of four items directed at protecting others exhibited medium internal consistency of 0.67, and acceptable (α = 0.71) if one item was excluded (caring for hygiene of oneself and her home). Principal component analysis on 3-item version extracts one principal component accounted for 65% of the variance in the dataset, with an eigenvalue of 1.96. For complete scales see Supplementary Material.

### Study Design and Procedure

Data collection for this cross-sectional study was initiated within 48 h after the first confirmed case of COVID-19 in Serbia was publically announced. As we were aiming to capture the relationship between observed variables and its stability through time, the survey was administered daily until the end of the first epidemic wave. This period of data collection lasted 10 weeks, and ended a week after the state of emergency was lifted in Serbia and the number of daily infections dropped significantly (at that time, we also observed a significant drop in the number of people responding to our survey; Supplementary Table 2). After that, the survey was again circulated in three more time frames, each roughly 2 to 3 weeks apart. These time frames were selected to capture significant changes in the progression of the epidemic (i.e., further rise of infections, peak of the second wave, then second improvement of the epidemiological situation).

The survey was hosted on a Slovenian local survey hosting platform 1ka.si that complies with national and European General Data Protection Regulation, guaranteeing participants' anonymity and secure handling of their personal data. We distributed the survey via our personal mailing lists, through colleagues, and using Facebook sharing and advertising.

Participants were firstly informed about the purpose of the study and the conditions of participation. After providing consent to participate, subjects were presented with the battery of tests which comprised scales described in the materials section, as well as scales assessing objective and subjective perception of knowledge about coronavirus, general trust in institutions, sources used for gathering information about the epidemic, and questions about personal general health status which will not be analyzed in this study. After completing the survey, participants provided their demographic information and were finally directed to the end page.

## Results

### Observed Constructs Over the Course of the Epidemic

**Figure 1:**
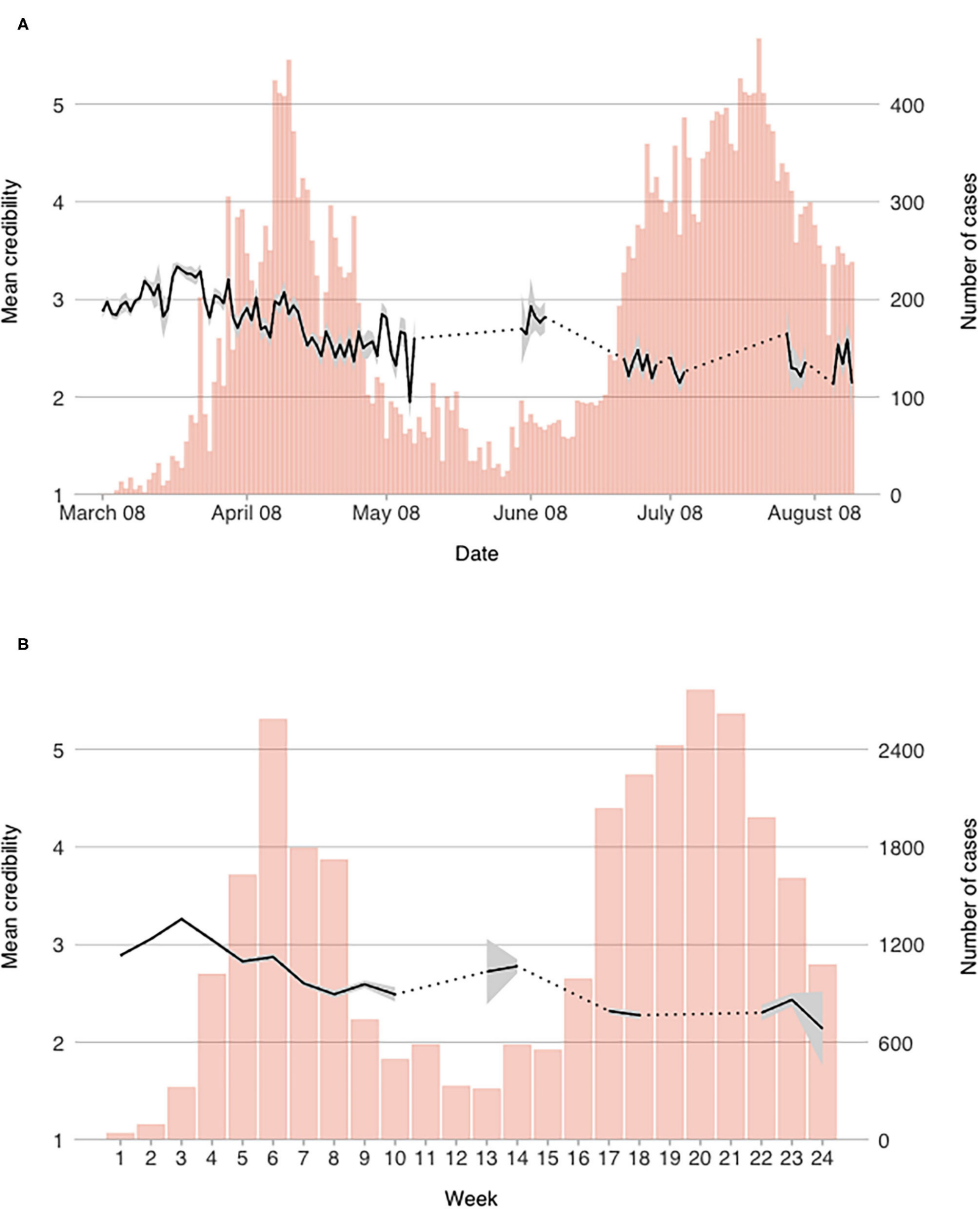
Mean perceived credibility of information scores on a **(A)** daily, and **(B)** weekly level.

**Figure 2:**
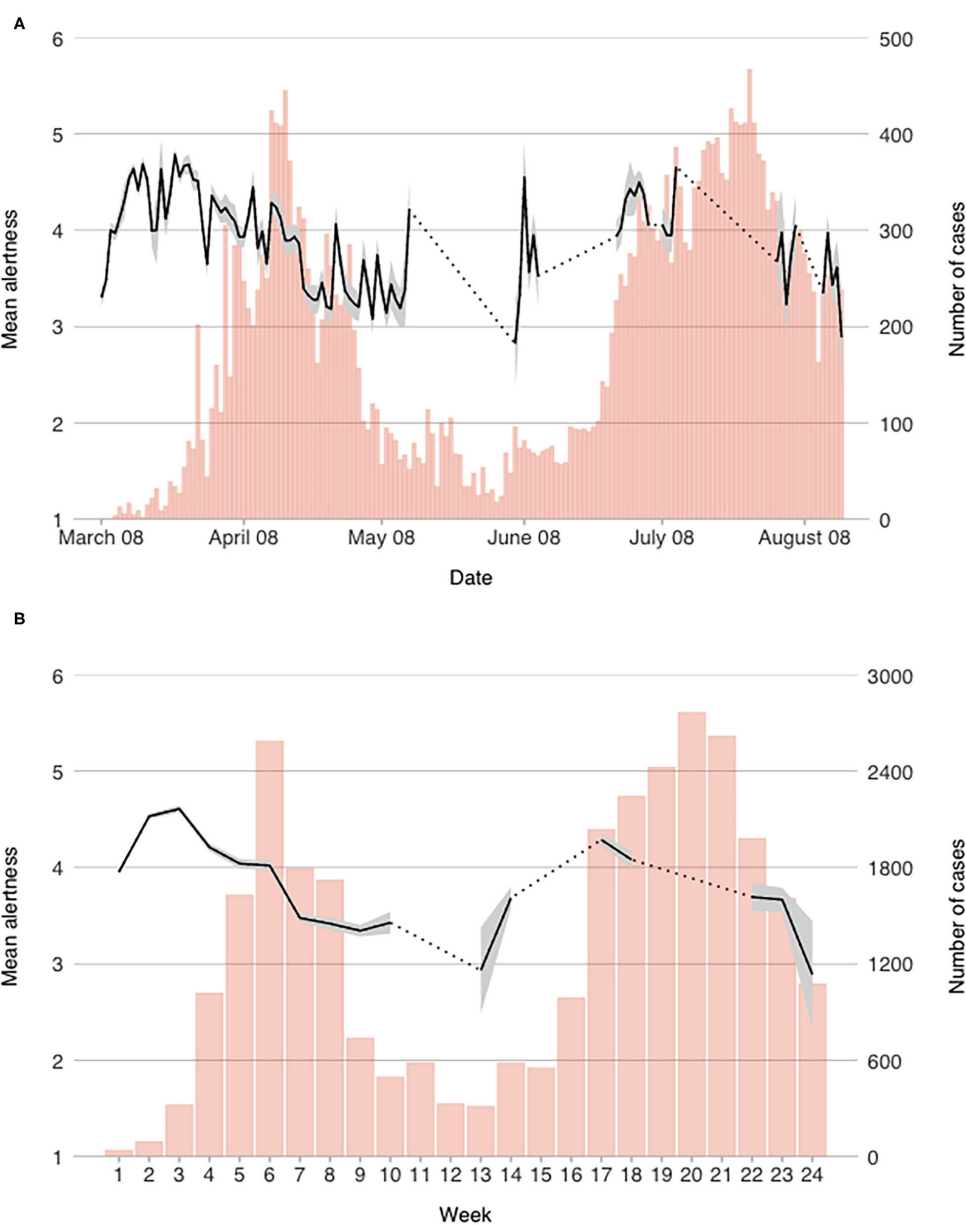
Mean alertness scores on a **(A)** daily, and **(B)** weekly level.

**Figure 3:**
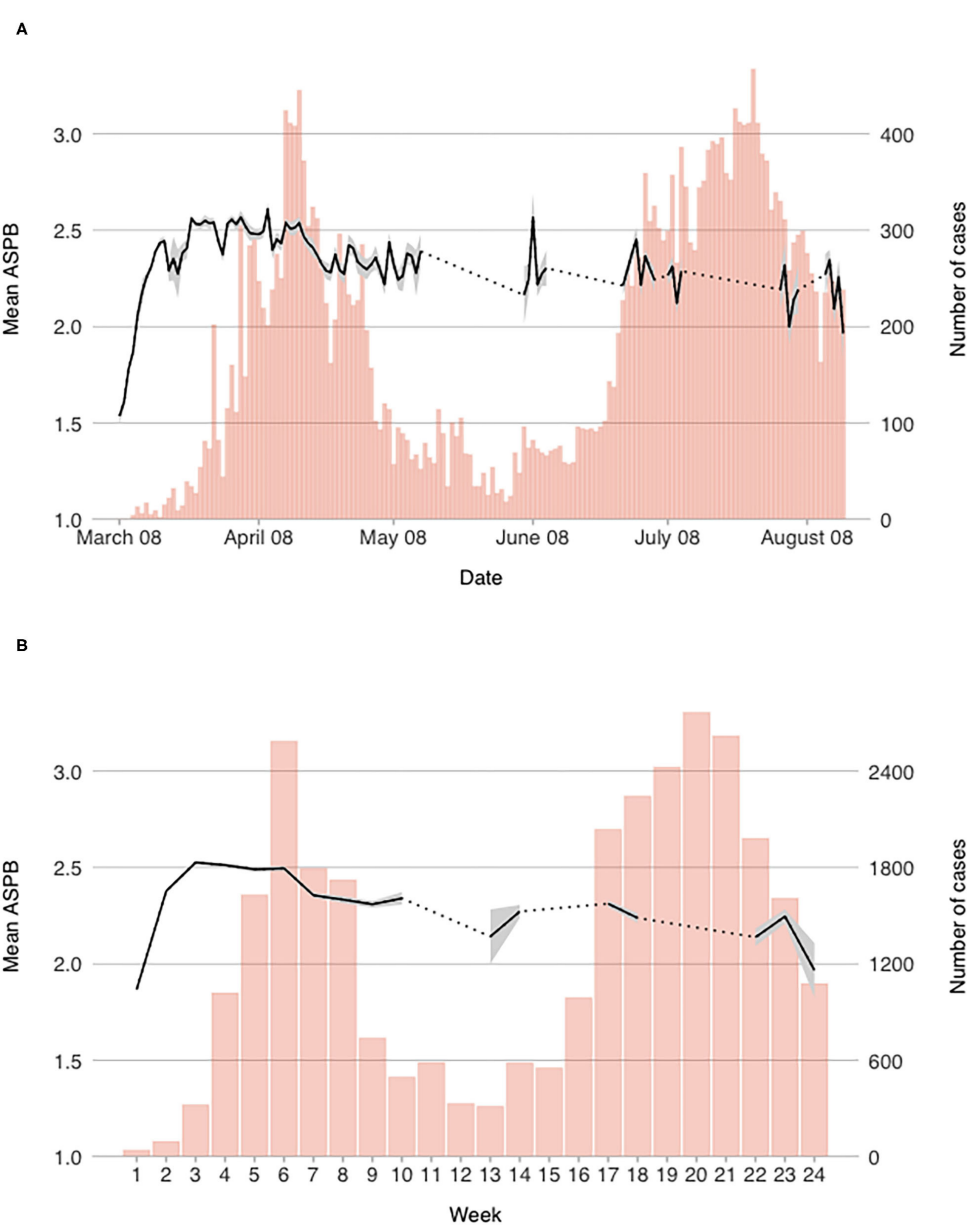
Mean reported engagement in actual self-protective behavior on a **(A)** daily, and **(B)** weekly level.

**Figure 4:**
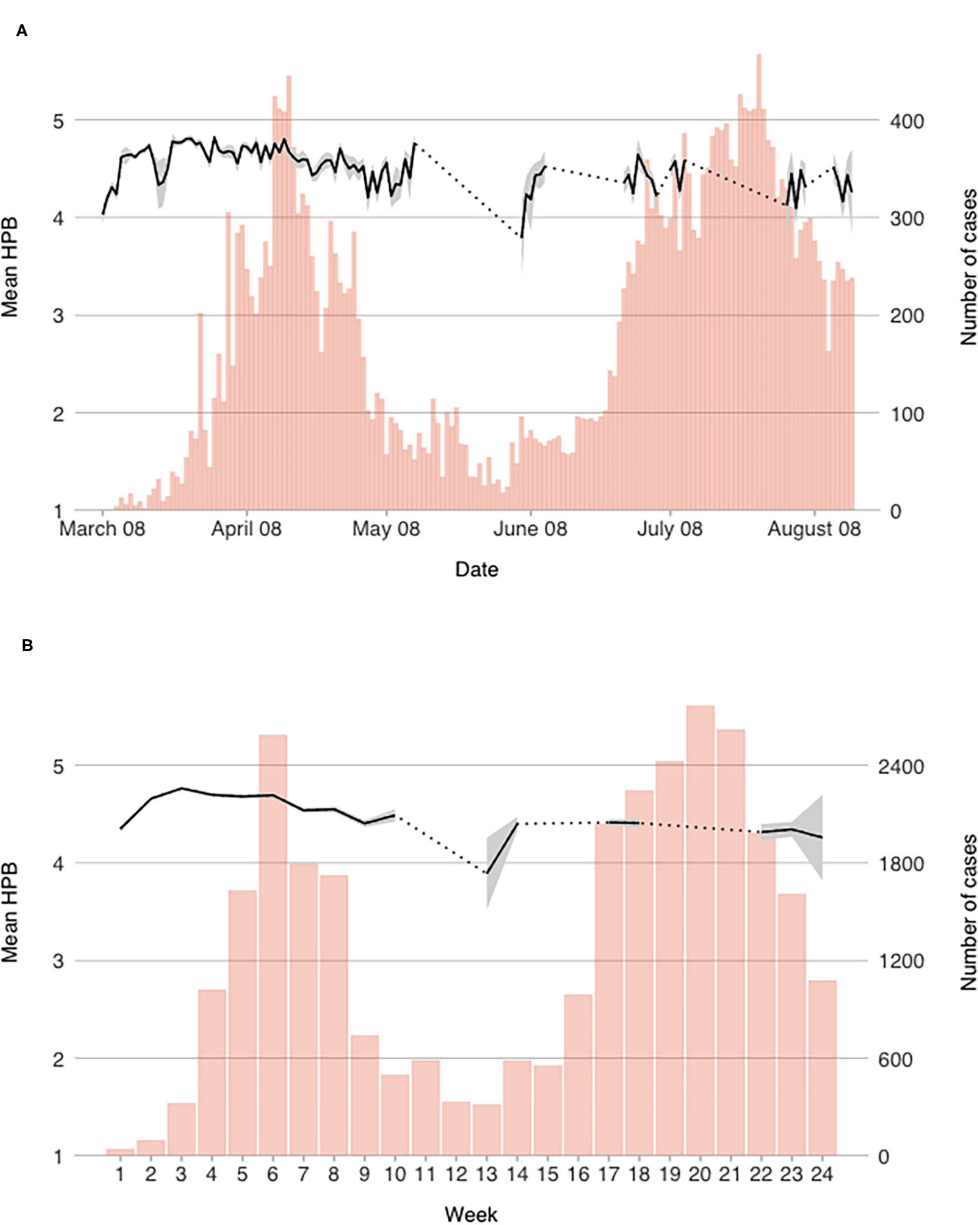
Mean reported engagement in hypothetical protective behavior on a **(A)** daily, and **(B)** weekly level.

For each of the measured variables, we computed daily mean scores. In order to observe more general trends that are less dependent on daily contextual changes, weekly scores were also computed. Both daily and weekly mean scores of the measured constructs are presented graphically in Figures 1–4 (for daily mean scores see also Supplementary Table 2).

#### Credibility of Information

Measure of PCI started out around midpoint and was increasing for the first three weeks (see Figure 1B). After that it started to diminish substantially; by week 4 it was back to the initial level and reached its lowest point during the first wave of the outbreak in week 8 (at the end of April). However, during the first wave, the absolute variability was within one point range and the mean daily score of PCI has never fallen below 2.3 out of 5. After the state of emergency was lifted, it remained relatively stable for another 2 weeks, but during the second wave, PCI decreased further.

Looking into day-to-day changes (Figure 1A), we can observe some notable jumps in the mean scores. The first is on March 21st and 22nd, when the number of participants was low in comparison to other days of the survey, and the mean score rises swiftly after that, reaching the highest mean daily score of 3.34 on March 24th. The next drop is observed around March 28th, when the Government of Republic of Serbia centralized the flow of information during the state of emergency (mean score dropped for 0.48 points between March 29th and March 31st). Mean PCI was gradually rising again until April 4th. Until April 16th the mean scores were relatively unstable and after that date, PCI was dropping until the measures were eased in the beginning of May (and the state of emergency was ended shortly after). The lowest daily score during the first wave was observed on May 13th, which was also the only day when the score dropped below 2 out of 5. Daily observations during the second wave are not numerous, but it seems that PCI remained lower than during the first wave as mean weekly scores continued to decrease in comparison to the week when the state of emergency was lifted (*M*_W9_ = 2.52, *M*_W12_ = 2.30).

#### Alertness

As with PCI, alertness was also on the rise in the beginning of the epidemic, though mean daily scores were initially higher (above 3.3 out of 6). After alertness scores reached peak on March 24th (*M* = 4.79), a notable drop in alertness was observed in the last two days of March. Even though two more short-term spikes were observed—the first on April 10th, and the second on April 28th—participants' alertness was generally diminishing since the end of March throughout the end of the first wave (see Figure 2). A notable spike in daily scores was observed when the number of cases started rising again in the beginning of June. During the second wave, a similar pattern was observed: means scores were rising during the first weeks and dropped significantly at the end of the second wave. As we did not collect data for every day during the second wave, we are not sure when the peak was reached, however it seems that mean alertness scores were lower than during the first wave.

#### Actual Self-Protective Behavior

Reported engagement in ASPB started out at midpoint of the scale (*M* = 1.54 on March 8th), but then steadily increased until week three when it plateaued (see Figure 3). After that it remained fairly constant for another 4 weeks, when it gradually started to decrease (with daily fluctuations). On a weekly level, one drop was observed around April 21st when the measures were eased for the first time (see Figure 3B). Still, the engagement remained stable after the temporal drop. During the second wave, our results do not point to any significant rise in ASPB, which ultimately dropped below the score 2 out of 3 in week 24. On a daily level, drops of lower magnitude can be observed (e.g., March 28th, April 7th, April 11th, April 15th, April 20th, May 1st), which mostly happened before or during the weekend lockdowns.

#### Hypothetical Protective Behavior

Conversely to ASPB, reported preparedness to engage in HPB started out relatively high (*M* = 4.20 on March 9th), and rose further until March 23rd (*M* = 4.78). The scores were consistently, albeit slowly, dropping after that date with temporal spikes—most notable on April 1st when the daily mean score of HPB peaked at *M* = 4.82—though the daily average has never fallen below 4.03 on a 5-point scale until the end of the first wave. At the beginning of the second wave, the scores were the lowest (below 4.0 in week 13; *M* = 3.89); after a surge in week 14, they again remained stable and as with alertness, lower than during the first wave.

Most notable drop in HPB scores was observed from April 16th, reaching a temporal low-point on April 22th at *M* = 4.43 (when measures were eased for the first time; see Figure 4A). Again, a notable drop was observed on a daily level around March 20th, when the number of daily participants was the lowest. While the scores on a weekly level were consistently dropping throughout the second half of April and in the beginning of May (see Figure 4B), scores on a daily level were relatively volatile in the first half of May. The same volatility can also be observed during the second wave, though less data was collected then.

### Relationship Between Variables and the Psychological Phases of the Pandemic in Serbia

While the changes in mean levels of observed constructs are not uniform across variables—they do not rise or decrease in unison, and some are relatively stable—there is some resemblance in their patterns of change. On days when the mean alertness scores were higher, so was the reported engagement in both ASPB (*r* between daily mean scores = 0.39, *p* < 0.001) and HPB (*r* = 0.54, *p* < 0.001). When mean daily scores of PCI were higher, so were mean scores of alertness (*r* = 0.52, *p* < 0.001), ASPB (*r* = 0.32, *p* = 0.002), and HPB (*r* = 0.52, *p* < 0.001). Furthermore, there was some overlap between both kinds of behaviors (*r* = 0.51, *p* < 0.001). We also observed whether the changes in the measured constructs were associated with daily numbers of infected or deceased, but those were notable only for PCI—on days with higher number of infected (*r* = −0.45, *p* < 0.001) and deceased (*r* = −0.51, *p* < 0.001), the participants perceived the credibility of information as lower. Their engagement in ASPB was slightly higher on days with higher numbers of infected (*r* = 0.25, *p* = 0.013), but was unrelated with daily numbers of deceased (*r* = 0.11, *p* = 0.271). Similarly, engagement in HPB and alertness were unrelated either to number of infected (*r*_HPB_ = −0.01, *p* = 0.936; *r*_alertness_ = −0.10, *p* = 0.333) or deceased (*r*_HPB_ = −0.07, *p* = 0.475; *p*_alertness_ = −0.09, *p* = 0.372).

Based on the changes in mean levels of the described variables as well as the external events, we divided the observed time frame into several phases. First, we divided the time until the state of emergency was lifted (which roughly corresponds to the first wave of infections) into three phases. The acute phase (March 8th—March 25th) is characterized by rising alertness, PCI and engagement in both ASPB and HPB and by first confirmed cases (and deaths) of COVID-19 in Serbia and the subsequent spread of the disease and introduction of ever-stricter official measures. During the adaptation phase (March 26th—April 21st) mean scores of alertness and engagement in protective behaviors ceased to rise, but remained fairly stable while the measures remained in effect and people were adapting to the new reality. The last phase, dubbed the relaxation phase (April 22nd—May 9th), could lastly be described by the diminishing number of new cases and eventual loosening of the official measures, while on the psychological level, alertness and PCI, were diminishing and people were engaging less in protective behaviors.

Further phases correspond to our data collection windows, as data was not collected on all days beyond the first wave. Phase four, the latent phase (beginning on May 10th) pertains to the time between the two waves, when the number of cases was comparably low, and the number of measures were relaxed. During this time, all the scores remained relatively low, as people were less alert and reported of lower engagement in ASPB and HPB. During the second wave of infections, the phases largely mirrored those of the first one. The latent phase is followed by the second acute phase (beginning on June 6th). Then, an increase in alertness, engagement in protective behavior, and PCI were observed. While the changes seem to occur over a similar time period (3–4 weeks), they were of a smaller magnitude. The second adaptation phase (June 26th—July 11th) is less clear: while PCI and HPB scores seemed to settle, alertness, and ASPB were more volatile, and larger daily changes were observed. As in the first wave, decreases of all scores were observed as the epidemiological situation improved and people, on the psychological level, entered the second relaxation phase (August 2nd—August 16th).

### The Role of Information Credibility and CAB Mediation Models

**Figure 5:**
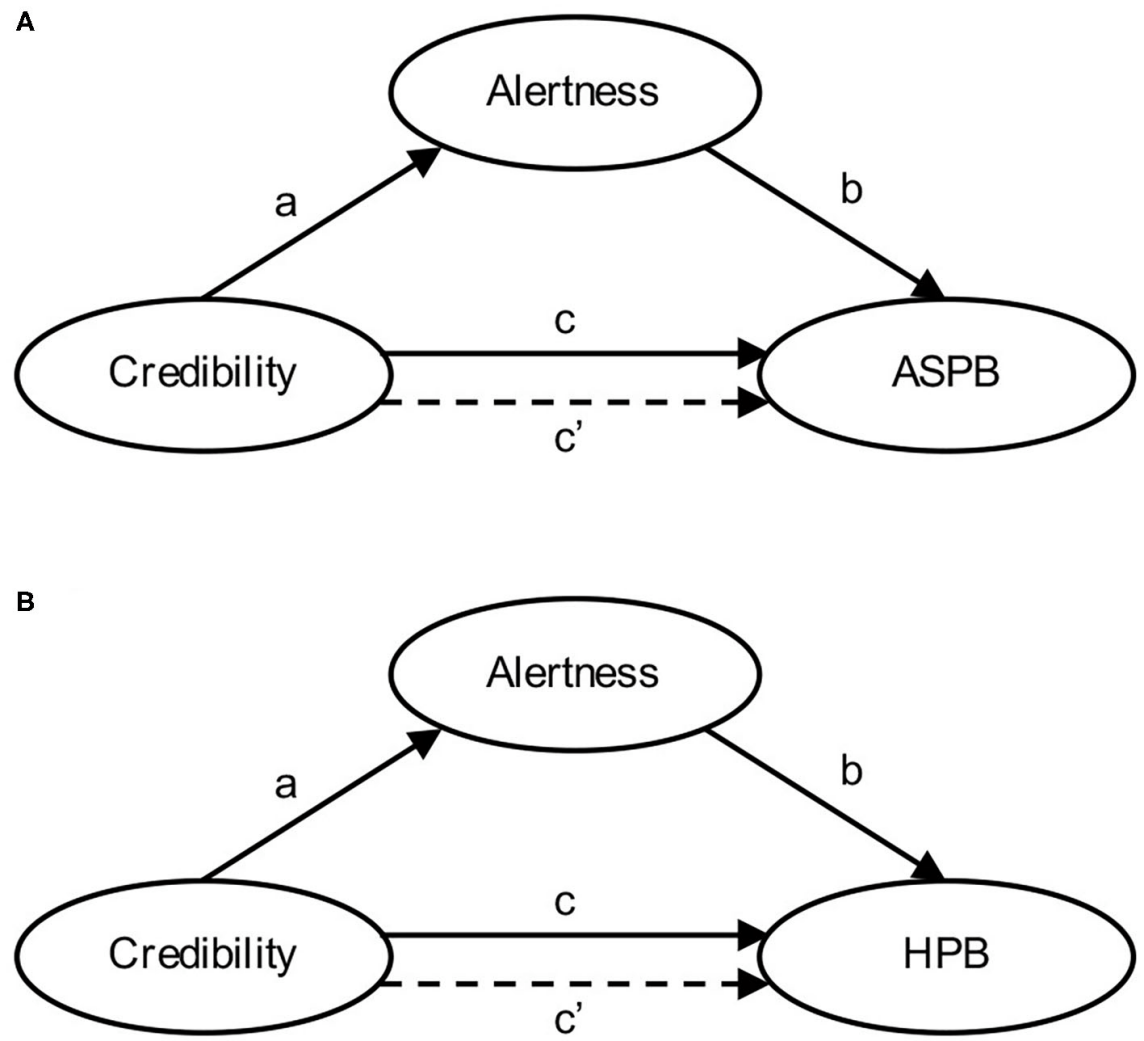
Proposed mediation models for predicting engagement in actual self-protective behavior (ASPB) and hypothetical protective behavior (HPB).

To explore how PCI is related to ASPB and HSP, two mediation models were tested (see Figure 5)—in both, PCI was a predictor, alertness was the mediator, and either ASPB or HPB were the outcome. Mediation was tested using R package lavaan (Rosseel, 2012) and confidence intervals were assessed using bootstrap. Each model was first tested for the entire study period, following the exploration of week-to-week (in the first wave), and phase-to-phase (throughout the pandemic) stability. We assessed the difference between total and direct effect using an online applet by Lee and Preacher (2013), and effect size was calculated as a simple ratio between indirect and total effects (Jose, 2013).

#### Actual Self-Protective Behavior

**Figure 6:**
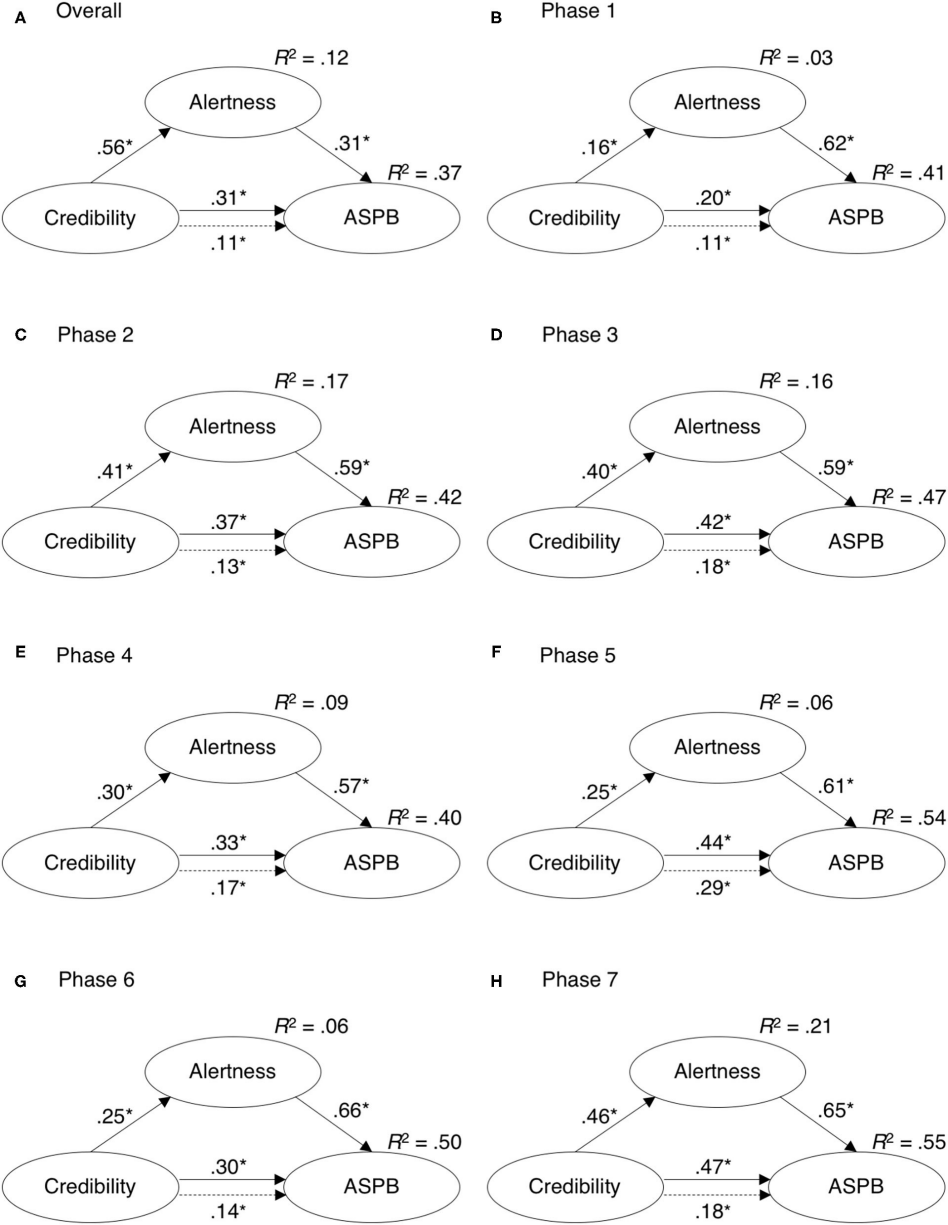
Mediation analysis results for predicting engagement in actual self-protective behavior (ASPB) from perceived credibility of information in **(A)** the entire duration of measurement and **(B–H)** in different psychological phases of the epidemic.

The overall relationship between PCI and ASPB was moderate (see Table 1), and partially mediated by the alertness scores. However, the strength of the relationship varied between weeks and phases of the epidemic (between *c* = 0.20 and *c* = 0.47). Predictive power of PCI on ASPB was weak in phase 1 (the acute phase), but higher in phases 3, 5, and 7, when the reported number of infected was lower. In all the phases, the effect was mediated by alertness: between 35% in phase 5 (*c* = 0.44, *c*′ = 0.29, 95% CI [0.19, 0.38]), and 65% in phase 2 (*c* = 0.37, *c*′ = 0.13, 95% CI [0.10, 0.16]). Overall, PCI and alertness explained almost a half of variability in ASPB scores. See Figure 6 for the graphical representations of results of the mediation analysis for each of the phases and for overall dataset.

**Table 1:** Mediation analysis results for Actual self-protective behavior (ASPB) credibility-alertness-behavior (CAB) model.

	**Model 1**		**Model 2**												
**Time period**	***c***	**95% CI**	***a***	**95% CI**	***b***	**95% CI**	***c'***	**95% CI**	a*b	**95% CI**	***tot***	**95% CI**	***Z***	***p***	***R***
Overall	0.31	[0.29; 0.32]	0.34	[0.32; 0.36]	0.56	[0.55; 0.58]	0.11	[0.10; 0.13]	0.19	[0.18; 0.21]	0.31	[0.29; 0.32]	13.70	<0.001	0.63
Week 1	0.06	[0.01; 0.12]	0.04	[−0.03; 0.10]	0.68	[0.65; 0.71]	0.04	[−0.01; 0.08]	0.03	[−0.02; 0.07]	0.06	[0.01; 0.12]	0.47	0.64	0.39
Week 2	0.19	[0.14; 0.25]	0.14	[0.08; 0.20]	0.59	[0.55; 0.63]	0.11	[0.06; 0.16]	0.08	[0.05; 0.12]	0.19	[0.14; 0.25]	2.07	0.04	0.43
Week 3	0.29	[0.23; 0.35]	0.32	[0.25; 0; 0.38]	0.51	[0.46; 0.56]	0.13	[0.07; 0.18]	0.16	[0.12; 0.20]	0.29	[0.23; 0.35]	3.80	<0.001	0.56
Week 4	0.39	[0.34; 0.44]	0.42	[0.36; 0.48]	0.57	[0.53; 0.61]	0.15	[0.10; 0.20]	0.24	[0.20; 0.28]	0.39	[0.34; 0.44]	5.61	<0.001	0.62
Week 5	0.38	[0.32; 0.44]	0.38	[0.31; 0.44]	0.61	[0.57; 0.65]	0.15	[0.09; 0.20]	0.23	[0.19; 0.27]	0.38	[0.32; 0; 0.44]	4.73	<0.001	0.61
Week 6	0.38	[0.32; 0.44]	0.43	[0.37; 0.49]	0.60	[0.55; 0.65]	0.12	[0.07; 0.18]	0.26	[0.22; 0.30]	0.38	[0.32; 0.44]	4.97	<0.001	0.68
Week 7	0.36	[0.30; 0.41]	0.38	[0.32; 0.43]	0.59	[0.54; 0.63]	0.13	[0.08; 0.19]	0.22	[0.18; 0.26]	0.35	[0.30; 0.41]	4.88	<0.001	0.63
Week 8	0.41	[0.34; 0.48]	0.37	[0.29; 0.45]	0.58	[0.52; 0.63]	0.20	[0.13; 0.26]	0.21	[0.16; 0.26]	0.41	[0.34; 0.48]	2.72	0.01	0.52
Week 9	0.47	[0.41; 0.53]	0.43	[0.36; 0.50]	0.59	[0.53; 0.64]	0.22	[0.16; 0.28]	0.25	[0.21; 0.30]	0.47	[0.41; 0.53]	4.76	<0.001	0.54
Week 10	0.33	[0.20; 0.45]	0.30	[0.15; 0.46]	0.57	[0.45; 0.68]	0.17	[0.03; 0.31]	0.17	[0.07; 0.27]	0.34	[0.21; 0.46]	1.61	0.11	0.51
Phase 1	0.20	[0.17; 0.24]	0.16	[0.12; 0.20]	0.62	[0.59; 0.64]	0.11	[0.08; 0.13]	0.10	[0.08; 0.12]	0.20	[0.17; 0.24]	3.84	<0.001	0.49
Phase 2	0.37	[0.34; 0.40]	0.41	[0.38; 0.44]	0.59	[0.56; 0.61]	0.13	[0.10; 0.16]	0.24	[0.22; 0.26]	0.37	[0.34; 0.40]	1.61	<0.001	0.65
Phase 3	0.42	[0.38; 0.46]	0.40	[0.35; 0.44]	0.59	[0.56; 0.62]	0.18	[0.15; 0.22]	0.23	[0.21; 0.26]	0.42	[0.38; 0.46]	7.06	<0.001	0.56
Phase 4	0.33	[0.20; 0.45]	0.30	[0.15; 0.46]	0.57	[0.45; 0.68]	0.17	[0.03; 0.31]	0.17	[0.07; 0.27]	0.34	[0.21; 0.46]	1.61	0.11	0.51
Phase 5	0.44	[0.32; 0.56]	0.25	[0.10; 0.40]	0.61	[0.51; 0.70]	0.29	[0.19; 0.38]	0.15	[0.06; 0.24]	0.44	[0.32; 0.56]	1.52	0.13	0.35
Phase 6	0.30	[0.24; 0.36]	0.25	[0.18; 0.32]	0.66	[0.62; 0.70]	0.14	[0.09; 0.19]	0.17	[0.12; 0.21]	0.30	[0.24; 0.36]	3.50	<0.001	0.55
Phase 7	0.47	[0.39; 0.56]	0.46	[0.36; 0.56]	0.65	[0.58; 0.71]	0.18	[0.10; 0.25]	0.30	[0.23; 0.36]	0.47	[0.39; 0.56]	4.08	<0.001	0.63

c—total effect (credibility ASPB), a—credibility alertness, b—alertness ASPB, c′–direct effect, a*b—indirect effect, R—ratio direct/indirect effect.

Looking into the relationship between variables on a weekly level during the first wave, we can see that the total effect was gradually rising (starting at *c* = 0.06 in the first week, and rising to *c* = 0.47 in week 9). The predictive power of PCI on alertness was initially non-significant (*a* = 0.04, 95% CI [−0.03, 0.10]), while it rose to *a* = 0.42 (95% CI [0.36, 0.48]) in week 4, and remained relatively unchanged until week 10. During the second wave, the predictive power was lower (between *a* = 0.25 and *a* = 0.30), but increased again in the last week of measurement (*a* = 0.46, 95% CI [0.36, 0.56]). The predictive power of alertness on ASPB was higher and comparably more stable throughout the course of the epidemic (between *b* = 0.51 and *b* = 0.68). With the exception of Weeks 1 and 10, when the change in path coefficients due to mediation was non-significant, the direct effect was consistently mediated by alertness.

#### Hypothetical Protective Behavior

**Figure 7:**
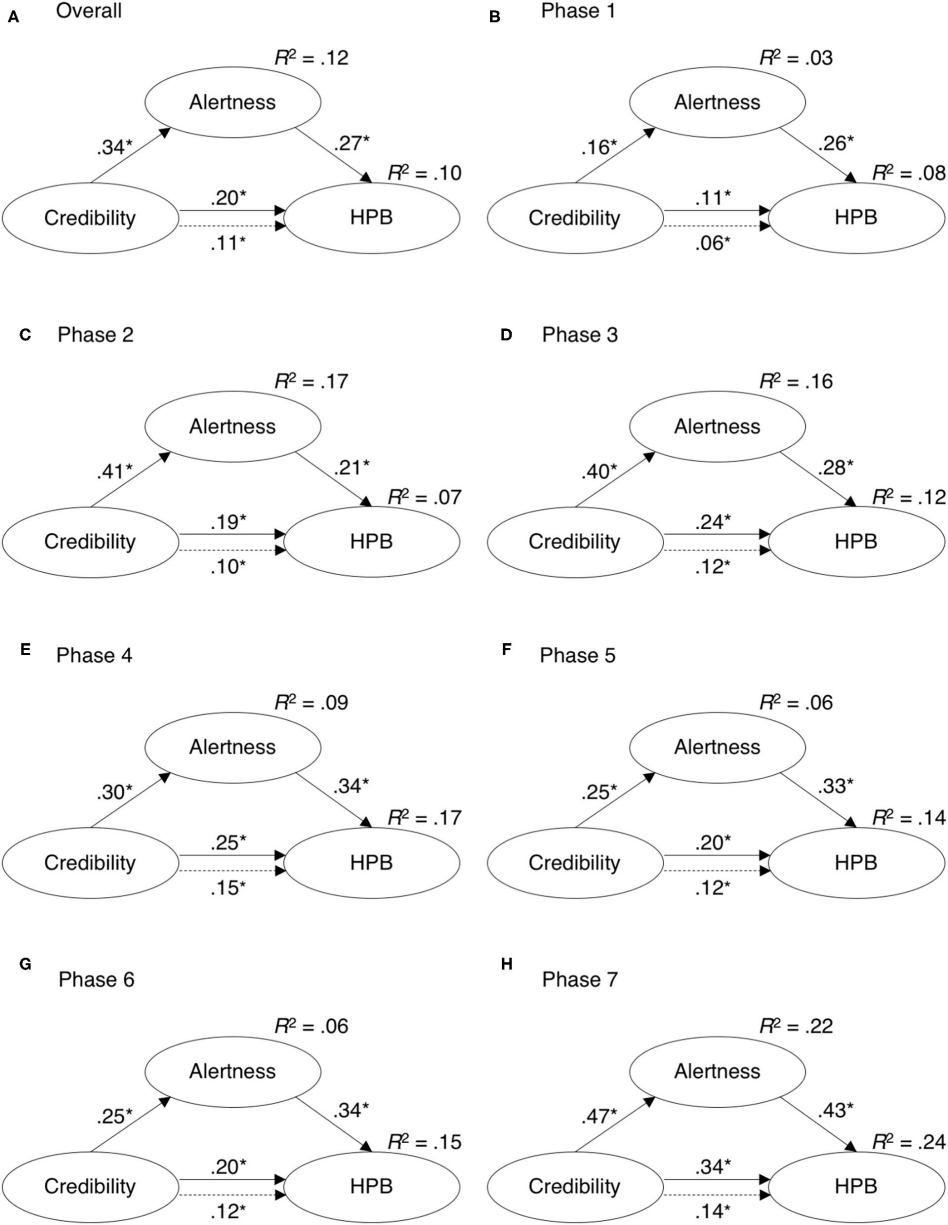
Mediation analysis results for predicting engagement in hypothetical protective behavior (HPB) from perceived credibility of information in **(A)** the entire duration of measurement and **(B–H)** in different psychological phases of the epidemic.

As with ASPB, the total effect of PCI to HPB and the a-path from PCI to alertness were initially weak (*c* = 0.02, *a* = 0.04) but they rose to moderate in week 9 (*c* = 0.27, *a* = 0.43). On the other hand, the predictive power of alertness in predicting HPB scores was relatively stable, albeit lower than with ASPB (see Table 2). Here, the mediation model accounted for less than a fifth of variability in HPB scores. However, variability of HPB scores was significantly lower in comparison to ASPB. The mediation models for each of the phases and whole duration of the pandemic are presented in Figure 7.

**Table 2:** Mediation analysis results for Hypothetical protective behavior (HPB) credibility-alertness-behavior (CAB) model.

	**Model 1**		**Model 2**												
**Time period**	***c***	**95% CI**	***a***	**95% CI**	***b***	**95% CI**	***c'***	**95% CI**	a*b	**95% CI**	***tot***	**95% CI**	***Z***	***p***	***R***
Overall	0.20	[0.18; 0.22]	0.34	[0.33; 0.36]	0.27	[0.25; 0.29]	0.11	[0.09; 0.13]	0.09	[0.08; 0.10]	0.20	[0.18; 0.22]	7.93	<0.001	0.46
Week 1	0.02	[−0.04; 0.07]	0.04	[−0.03; 0.10]	0.23	[0.17; 0.29]	0.01	[−0.05; 0.06]	0.01	[−0.01; 0.02]	0.02	[−0.04; 0.07]	0.21	0.83	0.56
Week 2	0.15	[0.09; 0.21]	0.14	[0.08; 0.20]	0.24	[0.17; 0.31]	0.12	[0.06; 0.17]	0.03	[0.01; 0.05]	0.15	[0.09; 0.21]	0.80	0.43	0.22
Week 3	0.08	[0.02; 0.15]	0.32	[0.25; 0.32]	0.17	[0.10; 0.25]	0.03	[−0.04; 0.09]	0.06	[0.03; 0.08]	0.08	[0.02; 0.15]	1.24	0.21	0.67
Week 4	0.16	[0.09; 0.23]	0.42	[0.36; 0.48]	0.17	[0.10; 0.23]	0.09	[0.02; 0.16]	0.07	[0.04; 0.10]	0.16	[0.09; 0.23]	0.16	0.12	0.44
Week 5	0.19	[0.13; 0.26]	0.37	[0.31; 0.43]	0.20	[0.13; 0.28]	0.12	[0.05; 0.19]	0.08	[0.04; 0.11]	0.19	[0.13; 0.26]	1.51	0.13	0.40
Week 6	0.21	[0.15; 0.28]	0.43	[0.37; 0.49]	0.20	[0.12; 0.28]	0.13	[0.06; 0.19]	0.09	[0.05; 0.12]	0.21	[0.15; 0.28]	1.75	0.08	0.41
Week 7	0.19	[0.13; 0.26]	0.38	[0.32; 0.44]	0.30	[0.24; 0.36]	0.08	[0.01; 0.14]	0.11	[0.08; 0.14]	0.19	[0.12; 0.25]	2.48	0.01	0.60
Week 8	0.25	[0.18; 0.32]	0.37	[0.29; 0.45]	0.28	[0.20; 0.37]	0.15	[0.07; 0.22]	0.11	[0.07; 0.14]	0.25	[0.18; 0.32]	1.77	0.08	0.42
Week 9	0.27	[0.20; 0.34]	0.43	[0.36; 0.50]	0.26	[0.19; 0.33]	0.16	[0.09; 0.23]	0.11	[0.08; 0.15]	0.27	[0.20; 0.34]	1.97	0.05	0.41
Week 10	0.25	[0.13; 0.37]	0.30	[0.15; 0.46]	0.34	[0.22; 0.46]	0.15	[0.04; 0.27]	0.10	[0.04; 0.17]	0.26	[0.14; 0.38]	0.90	0.37	0.40
Phase 1	0.11	[0.07; 0.14]	0.16	[0.12; 0.20]	0.26	[0.22; 0.30]	0.06	[0.03; 0.10]	0.04	[0.03; 0.05]	0.11	[0.07; 0.14]	1.61	0.11	0.40
Phase 2	0.19	[0.15; 0.22]	0.41	[0.38; 0.44]	0.21	[0.17; 0.24]	0.10	[0.07; 0.14]	0.08	[0.07; 0.10]	0.19	[0.15; 0.22]	3.57	<0.001	0.45
Phase 3	0.24	[0.19; 0.28]	0.40	[0.35; 0.44]	0.28	[0.24; 0.33]	0.12	[0.08; 0.17]	0.11	[0.09; 0.13]	0.24	[0.19; 0.28]	3,24	0.00	0.48
Phase 4	0.25	[0.13; 0.37]	0.30	[0.15; 0.46]	0.34	[0.22; 0.46]	0.15	[0.04; 0.27]	0.10	[0.04; 0.17]	0.26	[0.14; 0.38]	0.90	0.37	0.40
Phase 5	0.20	[0.07; 0.34]	0.25	[0.10; 0.40]	0.33	[0.20; 0.46]	0.12	[0.00; 0.25]	0.08	[0.02; 0.14]	0.20	[0.07; 0.34]	0.75	0.45	0.40
Phase 6	0.20	[0.14; 0.27]	0.25	[0.19; 0.32]	0.34	[0.27; 0.41]	0.12	[0.05; 0.18]	0.09	[0.06; 0.12]	0.20	[0.14; 0.27]	1.78	0.07	0.43
Phase 7	0.34	[0.23; 0.44]	0.47	[0.37; 0.57]	0.43	[0.33; 0.52]	0.14	[0.03; 0.25]	0.20	[0.14; 0.26]	0.34	[0.23; 0.44]	2.58	0.01	0.59

c—total effect (credibility HPB), a—credibility alertness, b—alertness HPB, c′–direct effect, a*b—indirect effect, R—ratio direct/indirect effect.

In weeks 1 and 3, the inclusion of alertness fully mediated the PCI-HPB link, and later the relationship was only partly mediated with alertness accounting for between 22 and 60% of the total PCI to HPB effect (note that the differences between *c* and *c*′ estimates were not all statistically significant). The mediation model was relatively stable over the proposed phases of the epidemic, however, both direct and total effects were doubled in later phases when comparing to the acute phase (see Table 2).

## Discussion

As in over 70 countries worldwide, the state response to COVID-19 outbreak in Serbia was immediate and broad. The first patient with COVID-19 in Serbia was registered on March 6th, and the first official state measures took place on March 15th, when the Serbian government declared the state of emergency, which was merely 4 days after the WHO declared pandemic. This was followed by relatively strict measures: instating the police curfew on March 17th, online schooling, and complete ban on leaving the house for senior citizens over the age of 65 (for an overview of the imposed measures, see Supplementary Table 1). The police curfew lasted on average for 12 consecutive hours on working days, while on weekends it was soon prolonged to 36 and even up to 84 h straight during the Easter weekend. Naturally, media outlets treated the pandemic as breaking news and sustained coverage began even before the first case of COVID-19 was confirmed in Serbia. Consequently, the COVID-19 related news comprised the vast majority of all daily media content. Through frequent official briefings and across various non-governmental informational sources, a myriad of changes to the measures, pleas for action and instructions were communicated to the public. In the present research, we were interested in how the public's perceptions of the information received and their alertness has changed, and how the PCI in different stages of the epidemic was related to actual and HPB.

### Psychological and Behavioral Unfolding of the Pandemic

As the pandemic is a lasting event, its progression was interrelated with changes in emotional and cognitive, as well as behavioral responses of the population. The PCI, alertness, and protective behavior, be it actual or hypothetical, was generally rising during the first 3 weeks of the outbreak. Following that rise, alertness, PCI, and reported intention to engage in HPB then gradually decreased over the following weeks, which is in accordance with previous findings regarding psychological responses to an epidemic (e.g., Cheng and Cheung, 2005; Bangerter et al., 2012). However, the scales and rates of decreases varied from close to negligible (HPB) to sizable (alertness and PCI), and are not in close accordance with the number of infections or deaths in a given day. We have to note here, however, that the correlations were observed on a daily level, but the effect of spike in infections or deaths on other constructs could be delayed and might only result in changes after some time, and could not be fully accounted for using a cross-sectional approach. Regardless, the observed patterns of correlations suggest that indeed some other (psychological) factors may also be in play when considering people's responses to the epidemic.

Alertness reached its peak on March 23rd right after the police curfew was prolonged to 12 h, and remained fairly unchanged until March 29th. This is in accordance with previously observed early emotional responses to COVID-19, exhibiting an abrupt increase in negative emotional responses during the first few weeks of the epidemic (Wang et al., 2020). Following this date (March 29th) on which officials announced that the full lockdown might be introduced, the alertness dropped significantly. However, over this and the following few days the mobile subscribers received a text message sent by the National Crisis Headquarters urging them to stay home as “we are coming close to Spanish and Italian scenario.” This was followed by a rapid increase in alertness scores. Although there were two more short-term spikes during the first half and the end of April, alertness was generally decreasing after the end of March, which is a pattern previously registered in studies on emotional responses to epidemics (e.g., Cheng and Cheung, 2005). By the 9th of May, the alertness was reduced to the level observed on the first day of the survey. During the second wave of infections, a similar pattern of changes was observed. In both cases, the observed spike in alertness occurred as the situation worsened, however it plateaued or started decreasing before the number of reported COVID-19 cases spiked in respective waves. This provides additional support for the robustness of the responses to the pandemic, and suggests the proposed psychological phases during the first wave of the epidemic could be mirrored to subsequent waves in terms of alertness.

Despite the obvious psychological pressure, the success of mitigating the spread of the infection depended on peoples' adherence to the measures. Our results indicate that evaluations of engagement in ASPB rose rapidly during the first 3 weeks, that is until the end of March, and remained at the consistent levels throughout the following 4 weeks. Actual self-protective behavior then decreased in the week from April 19th to 26th, which was during the last half of the Easter 84 h long police curfew, and simultaneously with announcements regarding the ease of protective and preventive measures and their actual waiving. However, the decrease in ASPB was then halted and mean scores remained stable until week 11. On the other hand, regarding HPB, initial increase in reported intent to engage in protective behaviors during the first 3 weeks was followed by a decrease in the ratings until the end of the survey. Again, a remarkable drop in HPB was observed during the seventh week (longest lockdown period along with the announcements regarding weaving of measures).

In short, and in line with the previous findings regarding decrease in commitment to various protective behaviors (Lau et al., 2003; Cheng and Cheung, 2005; Karademas et al., 2013; Yeung et al., 2017), people were less ready to adhere to preventive and protective behaviors as the epidemic progressed. Moreover, contextual changes, such as easing of the state protective measures or not being able to leave home for more than 3 days seem to have induced decrease in adherence to both actual and intended behavior. Here, we stress that our data do not allow us to argue if the behavioral change was solely due to one contextual factor or the other, or their combination, and that the changes in adherence to protective behavior were gradual and of a small magnitude. While high adherence to protective behavior could be a result of effective communication and people's high motivation as proposed in literature (e.g., Lewandowsky et al., 2013; Van Bavel et al., 2020), it could also be attributed to strict measures enforced by the government, leaving people very little space for exercising their behavioral differences. It is worth noting, though, that at all time points, individual variability in reported measures was greater than population-level changes over time, suggesting that even if people are mandated to do so, the adherence to measures is influenced by the individual differences.

### Perceived Credibility of Information

During an epidemic, public health authorities are expected to provide accurate information about the spread and effects of the outbreak in a timely manner (Tumpey et al., 2018; WHO, 2018). Aiming to persuade the citizens to change their behavior, it is important to note that the sources perceived as credible are also more persuasive (Petty and Brinol, 2008; O'Keefe, 2016). In the present study, PCI increased during the first 3 weeks, and following the peak, reached on March 24th, the PCI started to decrease. Ultimately, at the end of the data collection period in August, the registered mean evaluation of the PCI was even lower than at the beginning of the survey, which is in line with previous findings about the changing dynamic of people's trust in different sources of information during the outbreak of H1N1 in both China and in Switzerland (Bangerter et al., 2012; Yeung et al., 2017).

Moreover, at the end of March, the Serbian government announced centralization of authority over all information related to COVID-19 outbreak, and sent out the previously mentioned text message about the possible approaching of Spanish and Italian scenarios (see Supplementary Table 1). At the same time a journalist was arrested for publishing an alarming report on conditions in one of the health care centers. All the above was followed by a decrease in PCI scores. The lowest evaluations of PCI during the first wave were registered during week eight, between April 26th and May 2nd, when it was announced that the intensity of the state mandated measures will be significantly reduced (i.e., reopening of the majority of small private-owned businesses). Taking these situational and contextual factors into account, it seems that the PCI might not only depend on the source expertise and trustworthiness (Van Bavel et al., 2020), but also on the consistency of the news delivered from different sources. Additionally, perceived reasonableness of the content of the information (e.g., removing state mandated measures abruptly) might have also undermined credibility. However, as we didn't include the measure of perceived reasonableness of the content of the information provided during the epidemic, such a conclusion is to be tested in future studies.

### Promoting “Good” Behavior Through Information and Alertness

Besides observing the changes in PCI over time, we also focused on the role of PCI and its relation to intertwined both emotional and cognitive responses of the pandemic, as well as engagement in two forms of protective behavior. To test whether adherence to ASPB and HPB was related to the PCI and whether the relation is mediated by participants' alertness, we tested two mediation models, one for each type of behavior, in various time-periods. The models dubbed as CAB demonstrated relationship between PCI and ASPB was moderate and partly mediated by levels of alertness throughout the outbreak, which is in part supported by previous findings on importance of affective measures in promoting behavioral responses (Jones and Salathe, 2009; Liao et al., 2014). Moreover, as the total effect of PCI was gradually rising, the effect of alertness on ASPB remained fairly constant with the exception of a drop between weeks 1 and 2. This is consistent with some research on the role of cognitive measures (e.g., Liao et al., 2014), but inconsistent with observations by Leung et al. (2005a) who reported a drop in significance of affect-protective behavior link. Our findings might thus point to the fact that the alertness, as measured in the present study, might be more cognitive than an affective construct. Regardless, individual differences in alertness scores were a potent predictor of ASPB. Together with PCI, our mediational model accounted for nearly a half of variability in ASPB scores. This is especially noteworthy when accounting for relatively low degrees of freedom for people to exercise different behavior under state-mandated restrictions.

Furthermore, the cognitive nature of alertness may also be in accordance with a myriad of evidence suggesting that negative emotional arousal, although extensively used through fear appeals in campaigns aimed at inducing health related behavioral change, has limited and at times even counterproductive effects on behavior (Ruiter et al., 2014). On the contrary, *credible* information was also directly predicting ASPB throughout the epidemic, but especially when the epidemiological situation was better. This finding might be especially useful, as promoting protective behavior in those time periods is especially beneficial in preventing further outbreaks or mitigating their scale and unfolding. However, as some findings point to the fact that the positive effects of preventive measures such as social distancing and lockdown are only observed after 2–5 weeks after introduction (e.g., Cot et al., 2021), people need to consider the temporal focus in assessing the usefulness of the measures and choosing to adhere to them at present (e.g., Shipp and Aeon, 2019), which is not accounted for in our model. To improve it, but also to improve the promotion of protective behavior, it would thus be useful to empirically account for whether people resort to future temporal focused in future studies, and to test how their temporal focus is linked with scores on alertness, PCI, or ASPB.

Similar patterns to those described for ASPB were also observed in the second CAB model, predicting HPB. There, the effect of PCI on alertness was also rising during the progression of the outbreak, while both the alertness-HPB and PCI-HPB remained relatively stable beyond the third week of measurement. Still, the share of explained variance of HPB was lower than in the first CAB model. This might be counter-intuitive, as HPM might be less influenced by state-mandated measures and thus both PCI and alertness might have stronger effects. However, the variability in mean scores of HPB was lower than in ASPB, possibly due to ceiling effect.

While the mediation model was stable starting from the third week of the outbreak, both direct and total effects were doubled from the acute to adaptation phase, suggesting that some time might be needed for people to adapt to the situation and for the relations between variables to be fully established. Furthermore, these observed changes confirm the plausibility of the notion that, though timely and focused credible informing of the public is non-disputable imperative during the whole course of the epidemics (Reynolds and Quinn Crouse, 2008; Reynolds and Seeger, 2014), those messages do not fall on the same psychological ground during different psychological phases of the epidemic. This means that credible sources could take into account stages of psychological response to pandemics in order to effectively communicate mitigation measures. If we only take the overall CAB model, based on all entries regardless of the psychological stage, we can miss important information about the dynamics of relationships between different variables of interest.

In terms of temporal changes in the predictive power of both CAB models, our results deviate slightly from similar study conducted during the first 100 h of the outbreak in Slovenia (Lep et al., 2020), where PCI was found to be significant even in the earliest hours of the outbreak, but still support the importance of credible information throughout the course of pandemic in order to elicit high adherence to protective behaviors. Our results further show support for continued monitoring of various variables throughout the extraordinary events. Again we stress here that pandemics are longer lasting and dynamic events, and it is not surprising that the relationships between variables were not the same at the end of the outbreak when people know the measures, and have experience with the virus, as they were initially when information relayed by the media could be conflicting and ever-changing, when people were adapting to living under lock-down, and trying to assess various aspects of danger.

## Conclusions and Limitations

One of the main contributions of the present study is the systematic monitoring of various psychological perceptions and responses throughout the whole epidemic situation in a given country. While some studies are available that aimed at monitoring the unfolding of the COVID-19 outbreak, present study is the only one to our knowledge that comprises the whole duration of the state of the emergency in a single country and two full waves of the outbreak, thus offering information that expand on the findings of similar, albeit shorter studies on the dynamic of people's perceptions and responses (Sibley et al., 2020). Because we started gathering data only 2 days after the first confirmed case and continuing gathering until the end of official measures we had a unique opportunity to track changes in those perceptions and responses as well as their interplay. Such data thus offer a rich insight into what kind of interventions and support may be most useful in different stages of the outbreak. Moreover, as PCI not only proved to be an important predictor in mediation models in all stages of epidemic, but had increasingly stronger effect with the time passing, this suggests that effective communication is not only important in the early stages of the outbreak, but perhaps even more so once people's initial emotional reactions start to decrease. As PCI was relatively highly correlated with ASPB throughout the course of the epidemic, mean PCI scores were lowest after the first wave, when notable drops in ASPB were also observed. Lower ASPB scores could point to objectively lower risk when the epidemiological situation in Serbia improved, but there is no clear reason for PCI scores to drop at that time. In any similar situation in the future, it might thus be beneficial to ensure that PCI remains stable throughout the epidemic, and contributes to population wide adherence to protective measures.

As the study was conducted in Serbia, the results may not be easily generalized to other countries, especially because of the differences in applied governmental measures and people's perceived credibility of various sources. A caution is also warranted considering the *content* of credible information. Appeals to fear and alarming framing of the information might negatively influence one's awareness (Van den Bulck and Custers, 2009), while reassuring and solution oriented framing might have an opposite effect. All the while, both types of messages might be perceived as credible, which should be controlled for in future research when examining the role PCI has in changing alertness. However, as the dynamic of the observed variables was rather robust and generally in line with literature, we believe the findings on the role of PCI and individual changes in promoting protective behavior might translate to other contexts. The present study, however, only focused on the effects of official channels of pandemic related communication. In future studies, researchers should thus expand their scope and also tackle the potential effects of personal communications and the role of social networks in potential and actual behavior during the pandemic, and how they interact with the official discourse.

Our study also has some limitations in terms of sampling; as the data was collected online, the sample could be biased in terms of age and informational literacy, though it is of adequate range in terms of education of participants and the geographic distribution of population in the country. At the same time, the study design was cross-sectional and one should be cautious when making inferences from the results. As participants differed from day to day, presented results do not represent changes on the individual level, but rather capture broader changes in the society. Moreover, sample sizes varied from day-to-day, but also in different time periods (as time progressed, the recruitment got harder). In later stages of the study, it is also likely that the survey attracted a somewhat biased population (e.g., those who were more worried, more interested in the topic). As the context of the study deviated significantly from normality, measures used were not validated beforehand, which could cause concern in terms of validity. However, measures were used beforehand in Slovenia, where they exhibited adequate psychometric characteristics (Lep et al., 2020), and were tested again to ensure their validity both in terms of culture and situation (various stages of the pandemic). Moreover, the tested model of the observed measures does not disclose any information about possible concurrent relations of these variables with other susceptibilities and conditions developed over the course of time in the first 161 days of epidemic in Serbia. The intensive dynamics of changes in collected data could have been partially shaped by the delayed or cumulated effects of the e.g., mental fatigue for alertness, changes in engagement in protective behaviors due to denial mechanism, wide spreading of conspiracy theories about COVID-19, or simply after recovering from the infection, or avoiding or the decrease of informing about epidemics for perceived credibility. Though this calls for caution in interpreting the results, the mediational model was relatively robust, which indicates that credible communication does indeed contribute to the both types of protective behaviors.

Finally, our research points to some potential areas of future research. As the pandemic on a global level is far from over at the time we write this, the dynamics of people's perceptions and responses in the post-pandemic stage remains to be explored. While our results point to the importance of effective communication when the situation is improving, the question remains as how to effectively communicate relevant information to people about loosening the official measures in a way people will still comply with recommendations about protective behavior and thus prevent further infection waves. In order to address the aforementioned shortcomings of the cross-sectional research approach using self-report questionnaires it could also be beneficial to triangulate different data types and techniques such as self-reports, big data (e.g., activity on social media, media use, data on purchases, and mobility), official records on the spread of the disease, mathematical models derived from such data, web scraping (media content), etc. This would allow to us to consider, for example, the content of the news people rated as more or less credible, and also to validate their reports (e.g., are the reported changes in behavior mirrored in actual behavior). This might be especially interesting as with passing time, people in our sample seem to have accepted the “new normal” and were less prepared to follow any official guidelines, but also less prepared to participate in this type of study, while the data remains crucial for researchers and officials alike. At the same time, anecdotal observations point to the rise of alternative facts, fake news, and conspiracy theories. The fight against misinformation and effective motivation thus remain great challenges for politicians and professionals of various expertise.

## Data Availability Statement

The analyzed datasets for this study will be uploaded in one of the open source repositories, upon the ending of the project *Psychological profile of pandemics in Serbia*.

## Ethics Statement

All procedures performed in the study that involved human participants were reviewed and approved by the Ethics Commission of the Faculty of Arts, University of Ljubljana (no. 181-2020). Written informed consent for participation was not required for this study in accordance with the national legislation and the institutional requirements. The study was conducted in accordance with the 1964 Helsinki declaration and its later amendments and comparable ethical standards.

## Author Contributions

All authors listed have made a substantial, direct and intellectual contribution to the work, and approved it for publication.

## Conflict of Interest

The authors declare that the research was conducted in the absence of any commercial or financial relationships that could be construed as a potential conflict of interest.

## Abbreviations

ASPB: actual self-protective behaviors
CAB model: credibility-alertness-behavior (mediational) model
HPB: hypothetical protective behaviors
PCI: perceived credibility of information.
