# Supplementary material for: One Hundred and Sixty-One Days in the Life of the Homopandemicus in Serbia: The Contribution of Information Credibility and Alertness in Predicting Engagement in Protective Behaviors
Source: Front Psychol. 2021 Jul 5;12:631791. doi: 10.3389/fpsyg.2021.631791 (PMC8287004; doi:10.3389/fpsyg.2021.631791)
Supplement: Supplementary file 1 [file Data_Sheet_1.PDF]

Supplementary Material

1 Progression of the epidemic in Serbia

Table 1. *The dates and events throughout the two months of survey administration period during COVID-19 epidemic in Serbia*

Date	Event
6 March	First registered COVID-19 case in Serbia
8 March	Study starts
15 March	State of emergency declared
17 March	Curfew imposed (from 8 pm to 5 am)
20 March	First death caused by SARS-CoV-2
21 March	Curfew extension announced
22 March	Curfew extended (from 5 pm to 5 am)
24 March	First talks about the plan to open temporary accommodation and treatment facilities for COVID-19 patients
28 March	The government adopts a decision on centralized dissemination of information on all matters related to COVID-19 disease
29 March	President of Serbia announces that he may recommend the Government to impose an extended 24-hours curfew (full isolation)
	Temporary accommodation and treatment facilities for COVID-19 patients open
31 March	Crisis Staff sends the following text message to users of certain mobile operator: "The situation is dramatic. We are very close to the scenario we've seen in Italy and Spain. Please stay at home. Crisis Staff for control of COVID-19 disease".
	Citizens receive a message from an unknown source about possibility that twenty-four-hours quarantine will be imposed, which is quickly dismissed by officials as fake news
1 April	Journalist publishes an article on alarming situation in Clinical Centre of Vojvodina and she gets arrested on the same day with charges of false reporting and panic spreading
	Crisis Staff sends the following text message to users of certain mobile operator: "The situation is dramatic. We are very close to the scenario we've seen in Italy and Spain. Please stay at your homes. Crisis Headquarters for control of COVID-19 disease".
2 April	The arrested journalist is released from custody
	The government revokes the decision on centralized dissemination of information on all matters related to COVID-19 disease
	Curfew extended to weekends (from Saturday at 1 pm to Monday at 5 pm)
3 April	Decision to open temporary accommodation and treatment facilities for COVID-19 patients

**Date**	**Event**
4 April	Start of weekend curfew
5 April	Extended curfew
6 April	End of extended curfew (at 5 am)
10 April	Start of sixty-hours curfew (at 5 pm)
11 April	Sixty-hours curfew
12 April	Sixty-hours curfew
13 April	End of sixty-hours curfew (at 5 am)
17 April	Start of eighty-four-hours curfew (at 5 pm)
18 April	Eighty-four-hours curfew
19 April	Eighty-four-hours curfew
	Easement of measures starting from 21 April announced: curfew will be one hour shorter (from 6 pm to 5 am), citizens over 65 years of age will be allowed to leave their homes every other day after 6 pm, small private companies can open for business. This will soon (but at a later date) be followed by reopening of bars, gyms, etc.
20 April	Eighty-four-hours curfew
21 April	End of eighty-four-hours curfew (at 5 am)
	The announced easement enters into force
26 April	A decision is adopted that during May Day holiday the curfew will be from 30 April to 4 May
29 April	The President announces that May Day curfew will not be from 30 April to 4 May but from 30 April to 2 May, instead
30 April	Start of May Day curfew
1 May	May Day curfew
2 May	End of May Day curfew
4 May	Bars and restaurants reopen
	Intercity road and rail transport are re-established
	Public transport reopens in some cities
6 May	The Government adopts a decision on abolition of the state of emergency and the end of curfew
7 June	Decision on abolition of the state of emergency and the end of curfew enters into force
8 June	Public transport reopens in more cities
	Shopping malls reopen
10 June	The derby football match between Partizan and Crvena zvezda attended by 20.000 spectators
21 June	Parliamentary elections

2 Measures

2.1 Perceived credibility of information scale

Please rate to what extent do you consider that the information about coronavirus to be credible when provided by the following sources:

1. Journalists (for example news anchors. field reporters etc.)
2. Representatives of the Ministry of Health
3. Representatives of the Institute of Public Health of Serbia (Batut)
4. Representatives of the Medical Chamber
5. Doctors (for example when guest appearing in the news program)
6. Scientists (e.g. pharmacists. microbiologists. epidemiologists)

Participants rated each option on a 5-point Likert scale (1- Not at all credible. 5 – Completely credible).

2.2 Alertness scale

1. How worried are (were) you because of the coronavirus?

   a) Before coronavirus appeared in Serbia

   b) Today

   5-point Likert scale (1 – Not at all worried. 6 – Very worried)

2. How do you (did you) estimate the seriousness of the coronavirus infection?

   a) Before coronavirus appeared in Serbia

   b) Today

5-point Likert scale (1 – Not at all serious. 6 – Very serious)

3. Are you (were you) afraid of the coronavirus infection?

   a) Before coronavirus appeared in Serbia

   b) Today

   5-point Likert scale (1 – Not at all. 6 – Very)

4. How do you (did you) think about the coronavirus?

   a) Before coronavirus appeared in Serbia

   b) Today

   5-point Likert scale (1 – Not at all. 6 – Very often)

2.3 Actual self-protective behavior scale

Please rate to what extent the following statements apply to you today (since the first case of coronavirus infection was confirmed in Serbia)

1. I wash my hand more frequently
2. I don’t touch my face
3. I avoid any contact with other people. even from afar.
4. I don’t attend events where there are a lot of people.
5. I spend more time at home.
6. I follow the news more often.
7. Since the first case of coronavirus infection was confirmed. I have canceled my trip abroad.
8. Since the first case of coronavirus infection was confirmed. I have bought basic supplies (for example food) in order to have reserves.
9. Since the first case of coronavirus infection was confirmed. I have bought protective medical supplies (for example protective masks. disinfectants).
10. I avoid close contact with other people (for example shaking hands).

Participants rated each option on a 3-point Likert scale (Not at all, Partly, Completely).

2.4 Hypothetical protective behavior

If you were to notice today that you had symptoms associated with the coronavirus infection. how likely would you be to do the following:

1. Self-isolation (avoiding contact with other people)
2. I would avoid my family members
3. I wouldn't go to work

Participants rated each option on a 5-point Likert scale (1 – Definitely wouldn’t. 5 – Definitely would).

3 Daily means of the observed variables

Table 2. *Daily number of participants and mean daily levels of alertness, perceived credibility of information (PCI), Actual self-protective behaviors (ASPB), and Hypothetical protective behaviors (HPB)*

		Alertness		PCI		ASPB		HPB	
Date	N	M	SD	M	SD	M	SD	M	SD
03/8	137	3.30	1.12	2.88	0.75	1.54	0.44	4.03	1.15
03/9	196	3.47	1.32	2.97	0.77	1.61	0.48	4.20	1.06
03/10	276	4.00	1.33	2.85	0.81	1.78	0.49	4.31	0.96
03/11	267	3.97	1.31	2.84	0.88	1.87	0.47	4.24	1.03
03/12	116	4.13	1.43	2.94	1.00	2.06	0.47	4.62	0.69
03/13	43	4.31	1.23	2.97	0.65	2.18	0.46	4.64	0.60
03/14	228	4.54	1.27	2.88	0.86	2.26	0.44	4.65	0.62
03/15	396	4.64	1.19	2.99	0.82	2.31	0.40	4.62	0.63
03/16	380	4.41	1.19	3.01	0.90	2.39	0.35	4.67	0.61
03/17	256	4.69	1.16	3.19	0.89	2.43	0.39	4.69	0.63
03/18	242	4.52	1.23	3.14	0.88	2.44	0.36	4.74	0.65
03/19	50	4.00	1.33	3.04	0.94	2.29	0.40	4.57	0.61
03/20	20	4.00	1.58	3.15	0.96	2.35	0.51	4.33	1.09
03/21	24	4.64	1.52	2.82	1.10	2.27	0.62	4.37	1.22
03/22	64	4.12	1.45	2.90	0.93	2.38	0.39	4.51	0.78
03/23	58	4.39	1.45	3.23	1.03	2.41	0.40	4.78	0.58
03/24	262	4.79	1.12	3.34	0.75	2.56	0.29	4.77	0.60
03/25	254	4.56	1.21	3.30	0.86	2.53	0.31	4.77	0.50
03/26	217	4.67	1.27	3.27	0.96	2.53	0.34	4.81	0.49
03/27	138	4.68	1.26	3.26	0.97	2.55	0.34	4.81	0.45
03/28	150	4.52	1.40	3.22	0.94	2.54	0.34	4.75	0.61
03/29	183	4.51	1.31	3.29	0.96	2.54	0.35	4.77	0.57
03/30	153	4.04	1.51	2.97	0.98	2.44	0.41	4.66	0.69
03/31	114	3.65	1.40	2.81	1.00	2.37	0.37	4.57	0.73
04/1	146	4.36	1.46	3.04	1.02	2.53	0.32	4.82	0.44
04/2	217	4.27	1.42	3.02	1.05	2.55	0.35	4.69	0.65
04/3	179	4.19	1.53	2.96	1.10	2.53	0.40	4.66	0.72
04/4	103	4.23	1.51	3.20	1.03	2.57	0.35	4.68	0.68
04/5	94	4.15	1.37	2.82	0.99	2.52	0.40	4.67	0.70
04/6	91	4.09	1.66	2.71	1.06	2.49	0.40	4.55	0.84
04/7	179	3.93	1.51	2.83	1.04	2.48	0.38	4.73	0.61
04/8	190	3.93	1.46	2.91	1.04	2.48	0.41	4.72	0.65
04/9	158	4.15	1.45	2.79	1.07	2.49	0.42	4.66	0.70

Date	N	Alertness		PCI		ASPB		HPB	
		M	SD	M	SD	M	SD	M	SD
04/10	62	4.45	1.35	3.02	0.93	2.61	0.27	4.77	0.51
04/11	74	3.81	1.64	2.69	1.02	2.40	0.40	4.57	0.86
04/12	130	3.99	1.55	2.72	1.02	2.45	0.42	4.73	0.59
04/13	116	3.65	1.54	2.61	1.06	2.43	0.42	4.60	0.82
04/14	178	4.28	1.36	2.98	1.03	2.54	0.32	4.75	0.54
04/15	112	4.24	1.52	2.95	1.06	2.51	0.36	4.67	0.71
04/16	93	4.12	1.57	3.07	1.08	2.51	0.40	4.80	0.42
04/17	142	3.89	1.58	2.85	1.03	2.54	0.39	4.67	0.75
04/18	133	3.89	1.70	2.94	1.16	2.47	0.45	4.62	0.89
04/19	141	3.93	1.54	2.87	1.10	2.43	0.45	4.57	0.84
04/20	89	3.86	1.54	2.68	1.02	2.41	0.47	4.60	0.87
04/21	306	3.39	1.37	2.53	0.94	2.37	0.40	4.59	0.78
04/22	217	3.33	1.36	2.61	0.89	2.32	0.44	4.43	0.91
04/23	98	3.28	1.52	2.53	1.07	2.29	0.44	4.46	0.89
04/24	104	3.28	1.46	2.42	1.03	2.28	0.46	4.54	0.88
04/25	97	3.46	1.44	2.67	0.97	2.37	0.43	4.58	0.84
04/26	114	3.21	1.49	2.55	1.12	2.29	0.45	4.59	0.82
04/27	80	3.18	1.46	2.40	1.06	2.27	0.47	4.47	1.07
04/28	71	4.06	1.64	2.54	1.04	2.42	0.48	4.71	0.57
04/29	108	3.68	1.53	2.41	1.01	2.41	0.44	4.58	0.88
04/30	55	3.37	1.50	2.58	1.13	2.34	0.47	4.50	0.97
05/1	111	3.29	1.62	2.36	1.06	2.31	0.53	4.54	0.89
05/2	86	3.24	1.49	2.67	1.04	2.30	0.50	4.47	0.90
05/3	117	3.20	1.60	2.50	1.22	2.32	0.47	4.53	0.90
05/4	58	3.69	1.68	2.54	1.12	2.36	0.48	4.20	1.18
05/5	100	3.41	1.70	2.57	0.97	2.29	0.47	4.47	0.80
05/6	106	3.08	1.56	2.42	1.02	2.22	0.55	4.26	1.15
05/7	83	3.74	1.62	2.85	1.13	2.44	0.44	4.47	0.91
05/8	116	3.38	1.57	2.81	1.01	2.32	0.50	4.56	0.86
05/9	95	3.14	1.52	2.44	1.02	2.24	0.50	4.22	1.18
05/10	37	3.44	1.55	2.32	0.86	2.26	0.49	4.34	1.13
05/11	48	3.28	1.42	2.67	1.06	2.38	0.41	4.33	0.96
05/12	58	3.19	1.58	2.65	1.13	2.37	0.42	4.60	0.72
05/13	19	3.38	1.67	1.95	0.75	2.28	0.52	4.40	1.00
05/14	27	4.21	1.52	2.60	1.11	2.39	0.45	4.77	0.49
05/31	1	—	—	—	—	—	—	—	—
06/6	14	2.82	1.74	2.70	1.37	2.16	0.57	3.79	1.46
06/7	40	3.33	1.59	2.64	1.00	2.24	0.51	4.24	1.14

		Alertness		PCI		ASPB		HPB	
Date	N	M	SD	M	SD	M	SD	M	SD
06/8	17	4.55	1.53	2.93	1.21	2.57	0.51	4.19	1.33
06/9	65	3.57	1.64	2.82	1.08	2.22	0.50	4.44	1.04
06/10	48	3.95	1.57	2.76	1.00	2.28	0.45	4.44	1.01
06/11	29	3.53	1.69	2.82	0.86	2.30	0.50	4.52	0.87
06/28	80	3.93	1.53	2.39	1.01	2.21	0.45	4.36	0.99
06/29	100	4.02	1.64	2.22	0.81	2.29	0.52	4.43	1.09
06/30	77	4.32	1.50	2.37	0.85	2.38	0.44	4.25	1.06
07/1	33	4.43	1.64	2.48	0.99	2.45	0.37	4.65	0.89
07/2	65	4.36	1.59	2.27	0.86	2.22	0.48	4.54	0.83
07/3	148	4.50	1.52	2.43	1.08	2.37	0.41	4.43	0.91
07/4	146	4.37	1.63	2.19	0.87	2.30	0.51	4.39	1.04
07/5	75	4.05	1.65	2.33	0.98	2.24	0.53	4.22	1.07
07/7	1	—	—	—	—	—	—	—	—
07/8	83	4.06	1.63	2.41	0.94	2.27	0.44	4.49	0.83
07/9	80	3.95	1.72	2.25	0.75	2.31	0.46	4.57	0.83
07/10	102	3.95	1.76	2.14	1.00	2.12	0.57	4.27	1.09
07/11	51	4.66	1.53	2.26	0.84	2.29	0.54	4.59	0.81
08/2	13	3.67	1.91	2.65	0.96	2.19	0.35	4.12	1.17
08/3	22	3.98	2.11	2.29	1.10	2.32	0.52	4.44	1.12
08/4	37	3.23	1.67	2.29	1.09	2.00	0.56	4.10	1.05
08/5	52	3.73	1.74	2.21	0.95	2.14	0.57	4.48	0.83
08/6	26	4.05	1.59	2.36	0.88	2.19	0.63	4.32	1.20
08/12	29	3.35	1.90	2.13	0.88	2.27	0.58	4.52	0.83
08/13	82	3.97	1.64	2.54	0.91	2.35	0.48	4.38	1.09
08/14	60	3.43	1.88	2.34	1.05	2.09	0.58	4.16	1.28
08/15	35	3.61	1.64	2.59	1.08	2.26	0.52	4.43	1.04

Notes. PCI – Perceived credibility of information, ASPB – Actual self-protective behaviors, HPB – Hypothetical protective behaviors.
